# Rhizosphere Microbiome Modulators: Contributions of Nitrogen Fixing Bacteria towards Sustainable Agriculture

**DOI:** 10.3390/ijerph15040574

**Published:** 2018-03-23

**Authors:** Nicholas Ozede Igiehon, Olubukola Oluranti Babalola

**Affiliations:** Food Security and Safety Niche, Faculty of Natural and Agricultural Science, Private Mail Bag X2046, North West University, Mmabatho 2735, South Africa; igiehonnicholas@yahoo.com

**Keywords:** microbiome, next generation sequencing, plant yield, rhizobacteria, rhizosphere, sustainable agriculture

## Abstract

Rhizosphere microbiome which has been shown to enhance plant growth and yield are modulated or influenced by a few environmental factors such as soil type, plant cultivar, climate change and anthropogenic activities. In particular, anthropogenic activity, such as the use of nitrogen-based chemical fertilizers, is associated with environmental destruction and this calls for a more ecofriendly strategy to increase nitrogen levels in agricultural land. This feat is attainable by harnessing nitrogen-fixing endophytic and free-living rhizobacteria. *Rhizobium*, *Pseudomonas*, *Azospirillum* and *Bacillus*, have been found to have positive impacts on crops by enhancing both above and belowground biomass and could therefore play positive roles in achieving sustainable agriculture outcomes. Thus, it is necessary to study this rhizosphere microbiome with more sophisticated culture-independent techniques such as next generation sequencing (NGS) with the prospect of discovering novel bacteria with plant growth promoting traits. This review is therefore aimed at discussing factors that can modulate rhizosphere microbiome with focus on the contributions of nitrogen fixing bacteria towards sustainable agricultural development and the techniques that can be used for their study.

## 1. Introduction

The rhizosphere is the zone of the soil environment that is endlessly regulated by exudates, border cells and mucilages released by plant roots [1,2,3]. “Rhizodeposit nutrients, border cells, mucilages, and exudates produced by plants attract and serve as food for microorganisms that are present in the rhizosphere [3]”. It therefore means that plant’s root exudates can influence the diversity of resident microorganisms and invertebrates in the rhizosphere and these organisms can as well influence the plants by releasing regulatory substance. Hence, rhizosphere organisms are considered as a well-developed external functional environment for plants [4,5,6,7] and they are regarded as plant’s second genome [8]. Since plants are regarded as metaorganisms [9], understanding the actual contributions of rhizosphere microbiome (especially nitrogen (N) fixing bacteria) towards plant health and productivity is necessary.

Imbalance in nitrogen cycling, soil physicochemical properties, changing climatic conditions and abiotic stresses are the interwoven factors threatening sustainable agriculture. Sustainable agriculture depends on fertile soil, but land degradation and rapid desertification cause estimated loss of 24 billion tons of fertile arable land globally [10]. Recent investigations have demonstrated that plant host and its developmental stages play a role in shaping rhizosphere microbiome structure [11,12,13]. Also, comparison of the microbial diversity in different maize rhizosphere provided indication of host genetic influences on the richness of the rhizosphere microbiomes [12,14], but whether these disparities differentially have effects on plant health and development of the corresponding plant cultivar is yet to be known. This finding indicates that it may be possible to harness host plant genetic variation in a way that will allow the incorporation of rhizosphere microorganisms into plant breeding programmes so as to stimulate beneficial interactions between plants and microorganisms.

Moreover, factors such as soil type determine the composition of both endophytic and free-living rhizosphere microorganisms [15,16,17,18]. Many studies have shown that soil has a great effect on bacterial and mycorrhizal fungal structure in the rhizosphere [19,20,21]. In one of the studies, soil and cactus rhizospheric microbial communities were surveyed using culture independent techniques and it was discovered that microbial communities were shaped mainly by soil properties from different geographical locations followed by the cactus rhizosphere [21]. In a similar study, the actinobacterial populations in the rhizosphere of strawberry plants grown in different soils were more alike than the bulk soil were to each other, suggesting that plant is a stronger modulator of microbial richness than the soil type [22].

Other extrinsic factors like climate change and anthropogenic activities can also modulate the microbial communities in a specific plant host [23]. In particular, anthropogenic activity such as the use of N-based chemical fertilizer is associated with environmental destruction [24]. This factor has led to the reduction in land mass that can be cultivated for agricultural purpose. Yet, there is increase in food consumption as a result of the rising human population and this poses a great demand on large hectares of viable land for agriculture. This challenge can be overcome by harnessing rhizosphere microbiome (such as N fixing bacteria) for different agronomic purposes, especially as microbial inoculants. The idea behind this agricultural practice is to expressly decrease the use of chemical fertilizers which have a lot of negative impacts on the environment. Thus, the use of plant microbiome has the potential of minimizing environmental pollution, the occurrence of disease out-break, enhancing plant development, health and productivity. These microorganisms which can be applied on agronomic seeds singly or in consortium include endophytic and free-living root microbiome especially N-fixing bacteria that are known to fix atmospheric N for plant use. N is among the most important mineral nutrients needed for plant growth [24] and modern agriculture currently needs a safe and sustainable source of N and this can be achieved by the use of microbial inoculants.

Researchers have consequently been giving attention to endophytic bacteria because of their role in increasing N input to the soil [25]. These efforts have demonstrated that root nodules of crops contain endophytic bacteria including *Rhizobium* spp., non-nodulating strain of *Endobacter medicaginis*, *Micromonospora* spp., *Microbacterium trichothecenolyticumn* and *Brevibacillus choshinensis* [26,27]. Taxonomic distinctions among bacteria associated with the stem, leaves and nodules of legumes have also been unveiled using culture independent techniques [28] nevertheless little is known about endophytic bacteria associated with some agricultural crops [29].

Hence, the fundamental questions to better comprehend plants and their associated microbiomes are “who is there?”, “how do they respond to variation in environmental conditions?” and “do they impact plant health and growth?” Proffering solutions to the above questions will result in better understanding of how environmental changes modulate plant microbial composition; a prerequisite to detect if and how such microorganisms might be utilized in the future to enhance plant growth and crop yield especially in arable lands faced with environmental perturbations. Genomic studies of the plant associated microbiome are of utmost importance to provide answers to the abovementioned posers. DNA based analyses of each microbial species or meta-genomic profile of entire microbial communities reveal the composition and functional potential of plant rhizosphere microbiome [30]. Among the currently used molecular techniques, NGS techniques have the greatest impact on DNA and RNA based analysis techniques. NGS techniques help to find solutions to problems that could not be solved previously, due to financial and technical constraints. Hence, plant associated microbiomes can currently be studied at a speed cum depth as never before.

This review is therefore aimed at discussing factors that can modulate rhizosphere microbiome with focus on the contributions of N fixing bacteria towards sustainable agricultural development and the techniques that can be used for their study [31,32,33,34,35].

## 2. Modulators of Rhizosphere Microbiome of Agricultural Crops

**Figure 1:**
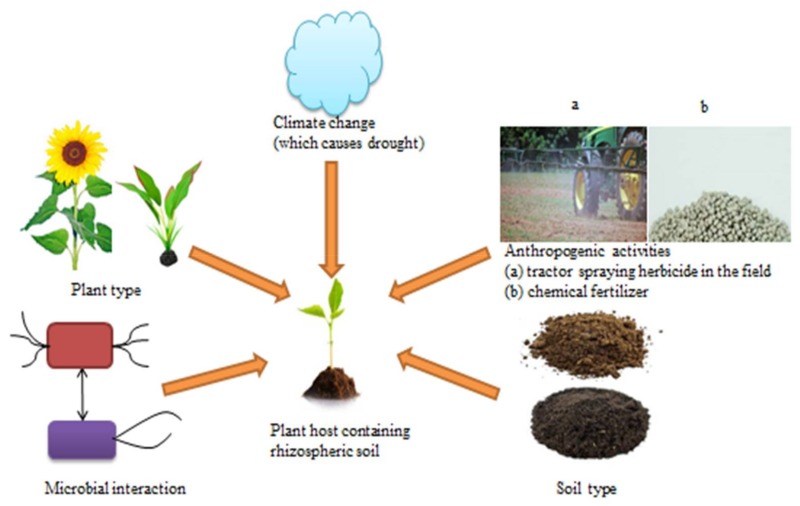
Rhizosphere microbiome modulators in a gricultural system.

Various factors such as biotic and abiotic parameters modulate or influence the microbial diversity and composition in the region surrounding the root (Figure 1). The level to which microbial communities are influenced by biotic and abiotic factors is not totally understood. Biotic factors such as soil type, plant host genotype/cultivar, climate change and anthropogenic activities are some of the abiotic factors that determine the rhizosphere microbiota composition [4,5,8,36,37,38].

Several studies have revealed that soil has a great effect on the structure of mycorrhizal fungal communities in the rhizosphere [21,39,40]. The physicochemical qualities of the soil influence plant health and type of root exudate release with the consequential effect on the structure of the rhizosphere microbial community. Sequence analysis of the bacterial community of the rhizosphere of different *Arabidopsis thaliana* cultivars revealed that soil type greatly affects rhizosphere microbial diversity [16,17]. The differences and resemblances observe in several studies could best be understood by viewing the microbial structure of the rhizosphere as emanating from a cascade of events. Firstly, the soil can be viewed as a seed bank of microorganisms [41] and secondly, the physicochemical characteristics of the soil coupled with bio-geographical activities determine the microbial assembly of the soil environment.

Soil can differ in structure, organic matter, pH, texture and nutrient status. These soil properties can select specific microorganisms by creating conducive environments that favour certain types of microorganisms and regulate the availability of roots exudates affecting selection of microorganisms by plants. In particular, soil pH and availability of nutrient such as carbon have been observed to affect the diversity of crop pathogenic nematodes, bacteria, fungi and beneficial microorganisms [42,43,44]. In some cases, soil properties may lead to soil type-specific composition of rhizosphere microbiome [45]. This was further confirmed by Gelsomino, Keijzer-Wolters [46] who demonstrated that bacterial community structures were alike in soils of the same type and Latour, Corberand [47] found that soil type affected the abundance and composition of *Pseudomonas* species in flax and tomato rhizosphere. This suggests that soil properties and soil type can determine the types of microorganisms that colonize the rhizosphere, and that different soil types can contain different microbial species (Figure 1).

Plant cultivar/genotype (Figure 1) influences the indigenous microorganisms present in the plant rhizosphere [14,40,48]. While physicochemical characteristics of the soil can influence the composition of soil microorganisms, plant root exudates are able to modify the rhizosphere environment that slowly alters the soil microorganisms to support the establishment of a rhizobiome [49]. These root exudates together with plant root immune system would further select those microorganisms that have the ability to colonize root surface (rhizoplane) and inner root tissue (endosphere). Microorganisms that colonize root tissues (endophytes) can have harmful or positive effects on plant species which eventually have feedback effects on rhizosphere microbiome [50]. Furthermore, certain metabolites liberated into the root region can elicit several responses in various soil microorganisms. In particular, flavonoids release from plants attract symbiotic microorganisms such as *Bradyrhizobium japonicum*, and disease-causing microorganisms e.g., *Phytophthora sojae*. The flavonoid naringenin produced by legumes activates germination of mycorrhizal spore and hyphal branching while the flavonoid catechin produced by *Combretum albiflorum* regulates quorum sensing [51,52]. Similarly, some defense metabolites (e.g., pyrrolizidine alkaloids) can affect the rhizosphere microbial structure by enhancing the growth of microorganisms that are able to break down these metabolites.

Recent study shows that variations between plant cultivar in a single gene could significantly affect the rhizosphere microbiome. The release of a single exogenous glucosinolates changed the microbial population on transgenic *Arabidopsis* roots [53] in which fungal and α-proteobacteria were predominantly affected, as revealed by denaturing gradient gel electrophoresis (DGGE). A study has also shown that the ABC transporter mutant of *Arabidopsis*, abcg30, produced root exudates containing high amounts of phenolic compounds and low amounts of sugars, which also gave rise to a unique rhizosphere microbiome [54].

Rhizosphere bacterial analysis using PhyloChip technique of three different cultivars of potato grown at two separate sites revealed 2432 operational taxonomic units (OTUs) and 40% of the OTU abundance was site-specific [55]. However, the abundance of 9% of the OTUs was cultivar-contingent in one or the other site, whereas only 4% of the OTUs had cultivar-contingent abundance in both sites. These outcomes demonstrate not only the significance of the soil in shaping rhizosphere microbial community structures, but also that certain microorganisms have a special affinity for specific plant cultivars. Amazingly, variations in abundance on the potato cultivars were observed for microorganisms belonging to the order Pseudomonales and families *Streptomomycetaceae* and *Micromonosporaceae*, which have been broadly studied for their capacity to control plant pathogenic microorganisms. This result further suggests that plant cultivar can influence the accumulation of bacteria that protect the plant against pathogens. Similarly, reports have revealed differences in the ability of wheat genotypes to assemble *Pseudomonas* species that produce antifungal metabolite 2, 4-diacetylphloroglucinol (DAPG), bringing about differences in disease suppressiveness [56,57]. Additionally, the quantity of antimicrobial substance produced on roots by certain biocontrol species differs between wheat genotypes [58]. Certain wheat genotypes were also found to differentially support biocontrol bacterial species indicating that there is a level of specificity in the association between plant cultivar and the microbial species in the rhizosphere soil environment [56].

It has been suggested that the modern way of plant breeding has probably not taken into consideration traits that are important for plants to serve as hosts to mutualistic microorganisms [4]. In an attempt to know the genetic components in plants responsible for establishing symbiosis with rhizobacteria, three “quantitative trait loci” in the genome of a tomato plant involved in suppressing disease caused by *Bacillus cereus* were detected. Furthermore, research on microbial diversity in the rhizosphere of maize showed that host genetic factor influences the composition of the rhizosphere microbiome [12,14].

In addition, microorganisms in the soil are a possible factor that influences the structure of the rhizosphere microbiome (Figure 1). Several studies have examined microbial colonization of the rhizosphere by intentionally coating crop seeds with certain microorganisms [4]. Previous investigations revealed that bacterial community on cucumber seedlings roots are more similar to the soil bacterial community than the seed coat bacterial biofilm, indicating that bacterial flora of the seed surface have little or no effect on the rhizosphere microbial structure. This may not be true for microorganisms present within the seeds, since study of endophytic bacteria of maize seeds showed the presence of one of the groups of the endophytic bacteria in maize rhizosphere [59]. Seed endophytic bacteria introduction into the rhizosphere suggests that plants could transfer certain microorganisms from generation to generation. Such carry-over effect on the structure of the rhizosphere microbiome has a significant effect on co-evolution of plant-microbial interactions in the environment [4].

### Other Rhizosphere Microbiome Modulators: Climate Change and Anthropogenic Activities

Microorganisms play important functions in all environments and so it is significant to understand how they respond to anthropogenic activities and climate change [60,61]. Climate change has different effects, ranging from local cooling to global warming, shifting vegetation zone and augmented extreme weather events and all these effects have indirect impacts on rhizosphere microbiome (Figure 1). Increase in carbon dioxide levels, a component that is alleged to be the key driver of climate change, could also directly affect rhizosphere interactions by changing root exudation patterns and soil food web structure and functioning [62,63,64]. The structure of the soil food web can play a significant role in ameliorating the impacts of extreme weather events [65]. Experimental modification of carbon dioxide, precipitation and temperature has also indicated that climate changes have significant effects on microbial composition and abundance in the soil [66,67]. Such transformations in microbial structure can similarly change the biogeochemical cycles/processes mediated by these microorganisms. Alterations in natural ecosystems caused by climate and anthropogenic changes can augment the impact of previously trivial biogeochemical processes or introduce new processes to the ecosystem [60].

An unraveled problem now is how microbial communities of the rhizosphere will respond to different facets of climate change. Rhizosphere microorganisms may have a greater ability to evolve than their host plants. In addition, as a result of their vast biodiversity, rhizosphere microorganisms may comprise of taxa that are acclimatized to warmer environmental conditions. The dispersal of soil microorganisms may also permit the immigration of microbial species from warmer environments into relatively cooler soil environments. Whether rhizosphere microbial composition under a particular climate change is dependent on spread of microorganisms or on their genetic acclimatization is an important question that needs to be addressed so as to know whether the microbial communities of the rhizosphere can cope with changes caused by global climate change.

In particular, climate change can cause abiotic stresses and some of the stresses associated with it are drought, cold and heat. It has been found that *Rhizobium* species can survive in dry environments, but their diversities are significantly lower in dry soil environments. Moreover, drought does not only affect the ability of *Rhizobium* species to fix N but also the growth and development of plant legumes. At times, it is difficult to separate the consequences of drought stress from heat stress as they both occur at the same time in semi-arid and arid regions of the world [68,69]. Suitable rhizobial species that can survive under drought environments in symbiosis with leguminous crops are of utmost importance [68] and therefore more research has to be focused in this area using modern state-of-the art techniques.

Furthermore, since the inception of the industrial revolution, anthropogenic activities in natural ecosystem have escalated as a result of the rising human population, pollution and ecosystem degradation and these have greatly affected soil microbial community structures. For instance, anthropogenic transformation of forest to farm lands resulted in the homogenization of the indigenous soil bacterial structure [70]. Thus, during the last 4 decades, the increasing awareness of the destructive impacts of human activities on the environments has triggered the enactment of laws to checkmate human behavior towards the environment. Though, the emphasis of the positive change in human behavior has been on the preservation of animals and plants while the negative impact of anthropogenic activities on microbial community has been relegated. This is a serious issue because microorganisms are the first responders to ecosystem disturbance and can either improve or buffer ecosystem shift [71]. Such ecosystem disturbance can occur as a result of herbicide and chemical fertilizer applications (Figure 1).

Herbicides are chemical compounds commonly used to control weeds in agriculture and many of them cause environmental contamination cum human health problems due to their toxicity and persistence in the soil [72]. Like chemical fertilizer, non-target impacts of herbicides on soil microbiome can be positive, negative or neutral [73,74,75]. reported that since herbicides kill plants, they can similarly kill or influence the activity of soil microbiome [74]. Soil microbial community is a crucial component of soil quality and thus, the effect of herbicides in the soil is very important.

The rhizobacteria community can be affected by the presence of herbicides such as glyphosate, frequently used where ‘genetically modified herbicide-tolerant’ crops are grown. The impact of glyphosate on rhizosphere microorganisms from agricultural crops like soybean and maize has been reviewed [76]. Certain herbicides like glyphosate move slowly in the soil and may adhere to organic matter and gradually degrade in soil and water [77,78] while others remain as recalcitrant in the soil or are leached into groundwater [79]. Although some herbicides persist in the soil, the herbicides glyphosate, atrazine and bentazon can be degraded by microorganisms. Atrazine-degrading microbiome might accumulate in the soil as a result of repeated applications and degradations of the herbicide [80]. But Toyota, Ritz [81] observed that atrazine and glyphosate might reduce soil microbial populations and enzyme activity while Ratcliff and Busse [82] observed changes in fungal and bacterial communities upon application of different herbicides.

Herbicides do not only affect microbial diversity, they also affect microbial functions (e.g., N fixation) in the soil. N fixation in legumes such as soybean can be triggered by symbiotic interaction between *Rhizobium* species and soybean roots and there is indication that the presence of sub-lethal amounts of glyphosate in growth media results in the accumulation of shikimate and decrease in *Bradyrhizobium japonicum* growth [83]. But, different *B. japonicum* strains exhibit different sensitivities to the herbicide [83,84]. Some species in the family *Rhizobiaceae* might breakdown glyphosate and utilize it as the main source of phosphorus in the presence of aromatic amino acids [85]. The impacts of glyphosate on nitrogen fixation by *Rhizobium* spp. in soybean have been studied in field and greenhouse experiments [86,87] even though the results as regards the effects of the herbicide on N fixation have been inconsistent. For instance King, Purcell [88] observed significant decrease in nitrogenase activity in glyphosate–resistant soybean when the herbicide was added three weeks after emergence and recommended that N fixing activity was more susceptible to glyphosate in the early stages of soybean growth. However, Powell, Levy-Booth [89] reported that there were no negative effects of the herbicide on N fixation under greenhouse experimental set-up. Also, soil microbial activity was affected when pure and formulated herbicides were applied to soils at rates greater than the recommended rate [90] and these effects of herbicides on microbial communities can either be short-term or long-term [91]. Furthermore, the level of chemical fertilizer application is also another important anthropogenic factor that modulates rhizosphere bacterial diversity of plants in the field [92]. Sequence analysis of bacterial communities of rice crops in fields amended with low and standard levels of N fertilizer showed that the rhizosphere microbiome was strongly affected by the level of N fertilizer. The abundance of OTUs in the genera *Bradyrhizobium*, *Methylosinus* and *Burkholderia* were higher in the rhizosphere microbiome from the field of the low level N fertilizer than standard level N fertilizer. On the contrary, the relative abundance of methanogenic archaea was higher in the field amended with standard level of fertilizer (SLF) than low level N fertilizer (LNF) field [93]. The genes *pmo*/*mmo* and *acdS* responsible for methane oxidation and plant interaction respectively were more abundant in rice rhizosphere microbiome grown in LNF field. Similarly, functional genes for the metabolism of sulphur (S), iron (Fe), aromatic compounds and N were significantly higher in the LNF rhizosphere microbiome [94], but, ^13^C-labeled methane experiment and quantitative polymerase chain reaction (qPCR) analyses for *mcrA* and *pmoA* genes coding for methyl coenzyme-M reductase and methane monooxygenase respectively indicated that methane oxidation was more active in the rice roots cultivated in LNF field than in those grown in SLF field [94]. These outcomes indicate that low-N fertilizer management is a crucial factor that modulates rhizosphere microbiome community structure and these coupled with other negative impacts of N based fertilizers necessitate the need for a more ecofriendly means of enhancing N level of agricultural land. This feat can be achieved by harnessing N-fixing endophytic and free living rhizobacteria.

## 3. Plant Endophytes and Their Ability to Fix Atmospheric Nitrogen

Nodule formation, which is a very efficient process of N uptake, has been reviewed extensively [95,96]. However, not all bacterial species can initiate nodulation because nodulation of plants such as legumes requires a complicated plant-microbe interaction. Certain bacteria penetrate roots via cracks initiated by “lateral root emergence” as well as wounds caused by “movement through the soil” [97]. These bacteria enhance plant development and play roles in N fixation [98]. Though there is no direct evidence that endophytes fix N in their plant hosts, the possibility of the process is broadly accepted. For instance, mutant strain of *Gluconacetobacter diazotrophicus* which lacks the capacity to fix N is not able to enhance its host plant growth as much as the wild type. The most studied endophyte that has the ability to fix N is *Pseudomonas stutzeri* A1501, which was first isolated in China [99]. The bacterium has possibly obtained genes coding for nitrogenase and enzyme that are adapted to different environmental conditions. The bacterium was studied so as to understand how nitrogenase activity and N fixation process are regulated and it was found that addition of ammonia to culture media stops the process of N fixation by N fixing bacteria. This is because the gene responsible for the expression of nitrogenase is down-regulated in that condition. For instance, the transcription of *nif* genes which are needed by free living organisms is repressed in the presence of ammonia. “Interestingly, *P. stutzeri* can switch between denitrification, nitrification, and N fixation under anaerobic, aerobic, and micro-aerobic conditions, respectively [100]”. Transcriptomic study has also unveiled a formerly unknown gene that plays role in N fixing process termed *pnfA*. *PnfA* is controlled by similar sigma factors as *nifHDK* (which code for nitrogenase). The expression of *PnfA* genes is not directly affected by mutation; however the mutant strain exhibits reduced nitrogenase activity particularly in micro-aerobic environment [100].

*Azoarcus* species are N-fixing plant growth promoting rhizobacteria (PGPR) and the wild type of *Azoarcus* sp. BH72 which inhabits kallar grass roots was able to enhance the dry weight of the grass cultivated in an environment deficient in N when compared with “*nifK* mutant strain of BH72”. Surprisingly, the bacterium can transform irreversibly from free living to endophytic forms and vice versa and as such it is not always feasible to re-isolate *Azoarcus* sp. BH72 endophytic colonies from roots [101].

*Rhizobium* sp. IRBG74 as well as *Azorhizobium caulinodans* which colonize rice roots have been isolated respectively from *Sesbania aculeata* and *Sesbania rostrata*. *Rhizobium* sp. IRBG74 has similarly been recovered from *Sesbania cannabina*, but being an endophyte, it does not have the potential to fix N since it lacks certain *nif* genes like *nifV*. *Rhizobium* sp. IRBG74 has now been re-categorized from the genus *Agrobacterium* to *Rhizobium* since it does not possess Ti plasmid, *fusA*, *rpoB* and 16S rRNA gene sequences. This bacterium possesses a sym-plasmid having *nifH* together with *nodA* genes [102] and it colonizes a wide range of *Sesbania* plants. Similarly, *A. caulinodans* ORS57 is able to colonize rice and fix N in endophytic form, however this bacterium should be tested with other plants species for its endophytic infection and N fixing abilities so as to know if this potential is unique to this plant sp. or is a common characteristic. “In order to determine whether it is the plant that initiates the N_2_-fixation in its bacterial symbiont (as regards nodulation), a common SYM pathway rice mutant should be tested for its ability to form endophytic symbiosis with ORS571 [103,104]”. It is also essential to distinguish identified multitude of genes of *Azorhizobium* responsible for plant infection, tolerance to stress and nodulation in order to ascertain those involved in *Azorhizobium*-*Sesbania* mutualistic interaction and rhizobial-legume associations.

*Herbaspirillum seropedicae* is an additional endophyte of plant roots that have been studied extensively and it infects the roots of rice, sugarcane and sorghum. It enhances the growth of its host plant by fixing N even in soil deficient in nitrogen and oxygen content [105]. *H. seropedicae* perhaps acquired its N fixing capability via “horizontal gene transfer” like other non-rhizobial strains. Like other disease-causing microorganisms of the genus *Herbaspirillum*, it is fascinating to know that *H. seropedicae* which is non-pathogenic has the entire genetic make-up for type I, II, III, V, VI and IV pili which it uses to facilitate communication with its host plant [106]. The type III is now known to be involved in the “initial signal communication” of *Bradyrhizobium elkani* and *Rhizobium* sp. NGR234 with their hosts [107].

Scientists’ attention on endophytes and their contributions to plant health and productivity should not only be theoretical and thus, these microorganisms can probably be utilized to improve nutrient absorption and plant diversity.

## 4. Rhizobiome as Plant Growth Promoters

Rhizobiome (which can be regarded as rhizobacteria) is a group of bacteria found in the rhizosphere that help to enhance the growth of their host plants. The rhizosphere has abundant microbial diversity and nutrients such as carbon substrates than the bulk soil. Microorganisms in the rhizosphere can be manipulated by wide range of complicated and highly monitored cell to cell interaction and by exploring signaling molecules to monitor their habitat and modify their activities. Rhizobacteria can affect nutrients absorption by plant roots directly or indirectly through nutrient mobilization/immobilization or alteration in root structure/physiology respectively. They can also enhance host plant nutrient status by increasing bioavailability of rhizospheric nutrients and improving other symbiotic interactions of the host [108]. Many microorganisms excellent at oxidizing manganese in the rhizosphere could therefore alleviate the toxic level of manganese in plants cultivated in oxygen deficient and saturated soils or enhance the manganese deficiency level in aerated soil containing high amount of calcium carbonate [2].

Rhizobacteria are a major component of PGPR, a terminology that was coined over 30 years ago [109]. PGPR are non-pathogenic bacteria that colonize plant roots and promote plant development and health by helping the plant to absorb more nutrients and control the proliferation of pathogens that would have been detrimental to the host plant [2]. Besides plant growth promotion, inoculation of rhizobacteria as a biofertilizer may enhances indigenous soil microbial diversity without leaving any negative effects in the soil unlike the conventional chemical fertilizers that have been reported to contaminate agricultural land upon application [110,111]. For instance, nitrates from chemical fertilizer can contaminate underground water and increase the risk of blue baby syndrome in new borne babies as well as stomach cancer in adults. Chemical fertilizer and pesticides can also have adverse effects on other environmental components such as surface water and soil fauna and flora.

It should be noted that some PGPR cannot be considered as biofertilizers. PGPR that enhance plant growth by eradicating pathogens are bio-pesticides and not biofertilizers. Amazingly, some PGPR seem to promote the growth of plants by functioning as biofertilizer and bio-pesticides. For instance, *Burkholderia cepacia* can destroy the pathogen *Fusarium* spp. and also promote maize growth under iron-deficient environments through siderophore production [112]. Moreover, siderophores as well as antibiotics produced by some species of rhizobacteria are pathogen-suppressing factors which could also be utilized for agricultural purpose. Both factors have microbial antagonizing properties and are able to stimulate systemic resistance [113].

However, some microorganisms such as arbuscular mycorrhizal fungi (AMF) present in the rhizosphere have been reported to have pesticidal traits [4,114]; a potential that could also help to nullify the negative impacts of chemical pesticide application and indirectly improve above and belowground plants’ biomass.

In particular, studies have shown how several rhizobiome influenced both above and below-ground biomass (Table 1) [115,116,117]. It is therefore desirable to harness a more ecofriendly, cost-effective and natural biological entities such as rhizobacteria and mycorrhizal fungi with soil-enriching, pesticidal and antimicrobial potentials for sustainable agricultural development [4,11].

## 5. The Effects of Rhizosphere Microbiome on Sustainable Agriculture and Food Security

The global world requires novel ideas for farming so as to be able to generate farm produce that can cater for world population of 6.9 billion. Actualizing food security which is the process of producing sufficient food and enhancing its quality to sustain the ever increasing population without undermining environmental protection is termed global green revolution [126]. Sustainable development in the area of agriculture is required to alleviate these issues. Development of agricultural practices (that are eco-friendly), natural resources and energy conservative strategy that guarantee food security are the critical aims of sustainable agriculture as reported by National Research Council [15]. It is the view of scientists that the most likely approach to actualize this objective is to replace hazardous inorganic fertilizers and pesticides with ecofriendly formulations of symbiotic microorganisms (such as *Bradyrhizobium* spp.) that have the potential to improve crop growth while providing protection from biotic stresses (such as plants pathogens and pests) and abiotic stresses (climate change/drought and environmental pollution).

Several studies on isolation, identification and application of microorganisms as an alternative method for chemical fertilizer utilization have been reported [127,128]. Enhancing the richness and abundance of soil microorganisms by this alternative method has also shown to improve plants health and yield [129,130,131]. In this case, the microbial cultures are mixed with chemical carriers using solid or liquid fermentation techniques. The microbial isolates are either incorporated to the plant in pure or mixed culture either via seed application, seedling dip, bio-priming or soil application. In addition to the use of individual microorganism, identifying suitable and functionally different microbiome and their usage for improving crop productivity is another huge and fundamental task to embark on since the entire microbiome is important, as it is described as the plant host second genome, “the metaorganisms” [15].

Hence, accomplishing food security is dependent on the enhancement of plant growth and diversity including seed yield right from the field and one of the ways this feat can be attained is through inoculation of agricultural crops (such as soybean) with rhizospheric rhizobacteria (e.g., *Rhizobium* spp.) as briefly discussed below.

### 5.1. Impact of Rhizobium Inoculation on Leguminous Crops Productivity

*Rhizobium* species are bacteria with the potential to reduce atmospheric N to ammonia for their host use through the formation of nodules on the roots or stems of leguminous plants [122]. This group of bacteria has been greatly studied due to their significance in agriculture and environment [132,133]. The amendment of seeds with *Rhizobium* species enhances seed protein, nodules formation and N absorption. In a review reported by Mfilinge et al. [122], soybean (*Glycine max* L.) inoculated with *Rhizobium* significantly increased the crop growth and yield constituents such as number of branches bearing pod per plant, total number of pod per plant and seed number per pod. Amendment of *Vicia sativa* L. (vetch) with *Azospirillum* in greenhouse and field experiments increased significantly N fixation activity, percentage N and N content. *Rhizobium leguminosarum* introduced into pea and lentil seeds was able to enhance pea nodulation, shoot/root diversity and yield of pea seed. Also, seedling height, nodule and shoot biomass of lentil were enhanced. There was also enhancement on nodulation of peanut treated with *Rhizobium* species while chicken pea treated with same species in greenhouse and field experiments resulted in significant increase in plant growth, root dry weight and number of nodules. Ravikumar [123] discovered that there was significant increase in the height, fresh weight, roots, nodules, leaves, shoots and pods number, pods length and seed weight of *Vigna mungo* and *Vigna radiate* inoculated with *Rhizobium* when compared to control experiments (Table 1). Height of soybean inoculated with *Rhizobium* in field experiment significantly increased and stem girth was also increased in greenhouse cum field experiments [134]. Similarly, in a study carried out by Nyoki and Ndakidemi [133], cowpea treated with rhizobial inoculants significantly increased the height of the crop when compared to the control counterparts.

These plant growth effects of *Rhizobium* species are enhanced when co-inoculated with other microorganisms. In co-inoculation, certain microorganisms function as helper microorganisms to enhance the effectiveness of the other microorganisms. Therefore, some bacteria can be co-inoculated with *Rhizobium* spp. that will not only enhance the effectiveness of the *Rhizobial* spp. but also improve crop productivity.

*Bacillus*, *Azotobacter*, *Pseudomonas*, *Enterobacter* and *Serratia* are some bacteria that have been successfully co-inoculated with *Rhizobium* spp. *Azospirillum* was observed to improve the productivity of legumes after inoculation [135]. Co-inoculation of *Azospirillum lipoferum* and *Rhizobium leguminosarum* bv. *trifolli* was observed to improve nodulation in white clovers [136]. *Azospirillum* was reported to increase the infection site for *Rhizobium* spp. leading to enhanced nodulation while inoculation of *Rhizobium* and *Azospirillum* was found to increase siderophore, vitamins and phytohormones production [137,138]. Amendment of common bean with *Rhizobium* and *Azospirillum* was similarly observed to improve N fixation in the crop [135]. *Azotobacter* was reported to be a prospective co-inoculant with *Rhizobium* that improved vitamins and phytohormones production resulting in increased nodulation [139].

*Bacillus* sp. was as well found to stimulate plant growth promoting (PGP) ability, nutrient uptake and crop yield [140,141,142] as increase in yield and root weight of chickpea was observed upon inoculation of *Rhizobium* with *Bacillus* spp. [143]. Improved N fixation and nodulation was detected when *Azospirillum* and *Bacillus* spp. were inoculated with *Rhizobium* in pigeon pea [135,144]. *Streptomyces lydius* WYEC108 interaction with *Rhizobium* was noticed to promote pea growth including nodule number, perhaps by *Streptomyces* colonization of root and nodule. Furthermore, *Enterobacter* species are PGP bacteria that enhanced the yield of nodules in plant upon co-inoculation with a species of *Bradyrhizobium*. There was increase in infection sites area and the number of root hair formed when *Medicago truncatula* was amended with *Pseudomonas fluorescens* WSM3457 and *Sinorhizobium* [145]. Also, shoot and root dry weight, nodulation, grain and straw yield, nodulation and phosphorus uptake were significantly enhanced in chickpea as a result of co-inoculation with *P. aeruginosa* and *Mesorhizobium* sp. [146]. Similar effects together with the antagonistic activities against *Rhizoctonia solani* and *Fusarium oxysporum* have been noticed on chickpea amended with a bacterial consortium of *Azotobacter chroococcum*, *Trichoderma harzianum*, *Mesorhizobium* and *P. aeruginosa* [147]. Although, different combinations of microorganisms have been explore, there is still a need for further research in this area [116]. *Rhizobium* introduction in leguminous crops is known for growth stimulation and is used as a substitute to the expensive conventional chemical fertilizers [122]. The use of suitable species as an inoculant in N depleted environments might be a better means to enhance the development and growth of legumes. Considering the relatively cheap rate of inoculation and the possible agricultural benefits, farmers are admonished to take advantage of these inoculants as bio-fertilizers on leguminous crops.

### 5.2. Impact of Rhizobium Inoculation on Mineral Nutrients Absorption by Leguminous Crops

The availability and absorption of mineral nutrients like nitrogen (N), phosphorus (P), magnesium (Mg), sulphur (S), calcium (Ca) and potassium (K) are very essential for the growth of plants especially in Africa where various cropping systems involving leguminous crops such as soybeans are practiced.

In particular, symbiotic interactions between legumes and *Rhizobium* spp. may be a major source of N in most cropping systems with about 80% of the needed N emanating from biological N fixation [148]. Indeed, Rudresh and Shivaprakash [149] reported that the amendment of leguminous seeds with rhizobial cultures enhances nodulation and N uptake. Studies on *Phaseolus vulgaris* indicated that N fixation from rhizobial inoculation contributed N equivalence of 20–60 kg of N per hectare in Brazil while Clayton and Rice [150] found that *Rhizobium* increased plant N from 19–42 mg per plant.

Also, P absorption is important for the development and proper functioning of plants and there is a large reserve of P in the soil environments, mainly in non-soluble forms that cannot be absorbed by plants and thus limiting plants’ growth. PGPR can solubilize the non-soluble forms of P through chelation, enzymatic activities and acidification [151,152]. The uptake of these mineral nutrients relies majorly on their concentrations, activities in the soil around the root region and their replacement ability in the soil. In legumes, this challenge can be surmounted by introducing *Rhizobium* species and essential mineral nutrients into the rhizospheric soil since *Rhizobium* and even other bacterial species such as *Bacillus*, *Burkholderia*, *Azospirillum*, *Pseudomonas* and *Erwinia* have been reported to have phosphate solubilizing potential [153,154].

Besides deficiencies in major nutrients, micronutrients such as zinc (Zn), boron (B), molybdenum (Mo) and iron (Fe) are also limiting nutrients that work against legumes productivity. It was reviewed by Mfilinge, Mtei [122], that the statistical significant increase observed for K intake was linked to *Rhizobium* inoculation into Pigeon pea (*Cajanus cajan* L. Millsp) and increase in the amount of nutrient in the environment increased the chance of plants uptake. Study carried out by Makoi and Bambara [155] on the impact of *Rhizobium* strains on mineral nutrient absorption by *Phaseolus vulgaris* showed significant increase in the uptake of P, K, Mg, Ca and S in the entire plant parts. It was reported by the author that even though the concentration of P and K skyrocketed in the root region due to *Rhizobium* introduction, the increase was only significant in the greenhouse experiment and not in the field condition. *Rhizobium* inoculation enhancement of micronutrient (such as Mn, Fe, Cu, Zn, B and Mo) uptake in the shoots, roots, pods and the entire plant with the exception of Mo intake in the roots has been reported. *Rhizobium* has also been shown to cause significant increase in Ca and sodium (Na) content and the pH of the soil [156]. The uptake of Zn, Fe, Mn and Cu by cowpea was significantly different between treatments amended with *B. japonicum* and P under greenhouse and field conditions [133]. *B. japonicum* caused significant increase in the intake of Zn, Fe, Cu and Mn in soybean (*G. max* L.) under greenhouse condition while uptake of Fe, Cu and Mn increased significantly and that of Zn decreased under field condition [134].

There is however limited information on the roles of different rhizobial species of legumes on the bioavailability of other mineral nutrients in bean cultivars especially in Africa. Similarly, the impacts of *Rhizobium*, P and K interaction have not been studied in detail and it is therefore important to study the potential role of *Rhizobium*, P and K on the bioavailability of other mineral nutrients in leguminous crops such as soybean grown in each ecological zone in Africa.

### 5.3. Impact of Rhizobium Inoculation on Chlorophyll Concentration and Photosynthetic Activities of Leguminous Crops

The crucial regulating element for plant growth is N due to its limited availability and also beans require this element more than other mineral nutrients. N is a component of most organic compounds such as proteins, growth regulators and chlorophylls. N deficiency can lead to stunted growth, yellowing of leaves and reduced branching in beans. N is one of the monomeric units of proteins and is greatly required for the entire enzymatic process in the cells of plants. It is also present in many vitamins and chlorophyll and plays a role in photosynthetic process [134]. In a study carried out in greenhouse and field environments, the chlorophyll content of the leaves of common beans significantly increased upon inoculation with rhizobial species. There was also significant increase in the photosynthetic activities of plant amended with rhizobial strains by 140 and 80% for greenhouse and field experiments respectively compared to control experiments [122].

It has similarly been reported that soybean amended with *B. japonicum* showed increase in chlorophyll content and growth indices such as height of plant, leaves number on a plant, stem width, day’s number to 50% flower and pod development as against the control counterparts [134]. Upon studying the impacts of P and *B. japonicum* on cowpea (*Vigna unguiculata* L.) growth, it was discovered that the chlorophyll content of the leaf of cowpea significantly skyrocketed for treatment inoculated with *B. japonicum* in the field. There is however little information on the impacts of K, P and *Rhizobium* inoculation and their interactions on formation of chlorophyll in *Phaseolus vulgaris* in Tanzania which is a gap that needs to be covered research wise [122].

## 6. Nexus of PGPR, Fe Acquisition, Plant Productivity and Pathogens Eradication

Besides inorganic P and N, Fe is an additional mineral nutrient that plants can obtain through symbiotic interactions with soil rhizobacteria. Although, ferric ion (Fe^3+^) is more available in the soil, plants usually prefer to absorb Fe in the form of reduced ferrous ion (Fe^2+^) [157]. Plant release phyto-siderophores and chelators which adhere to Fe^3+^ and helps to retain it in soil solution. The chelators release the Fe^3+^ to the surface of the root where it is converted to Fe^2+^ and absorbed immediately while phyto-siderophores secreted by grasses are absorbed with Fe^3+^ across plasma membrane [158].

On the other hand, some PGPR are able to sequestrate the insoluble form of Fe from the soil with the aid of siderophores making it available for the host plant [99] and there is indication that a couple of plants can absorb bacterial Fe^3+^-siderophore complexes [159] for their growth. However, the importance of bacterial Fe^3+^-siderophore absorption to plants’ iron nutrition is controversial. Some researchers reported that the contribution of Fe from this source to plants’ overall Fe requirement is small [160] while others gave credence to their important and vital contributions especially in calcareous soils [161,162]. Bar-Ness et al. [163], who previously accepted the idea of bacterial siderophore uptake by plants [159], reported that two bacterial siderophores namely ferrioxamine B and pseudobactin did not effectively provide iron for plants.

The sequestration or acquisition of Fe through PGPR siderophores decreases the bioavailable Fe in the rhizosphere and as such affect the growth of fungi that might be pathogenic to the plant [164,165]. In Fe-deficient soil, plant is more productive in microorganism-rich soil than in soil devoid of microorganisms, buttressing the fact that PGPR help the host plants in acquiring this limited mineral nutrient [99].

## 7. New PGPR That Are Related to Human Opportunistic Pathogens

There are some rhizospheric microorganisms that have the potential to cause opportunistic infections in humans. Berg and Eberl [166] suggested that different bacterial genera such as *Enterobacter*, *Staphylococcus*, *Herbaspirillum*, *Pseudomonas*, *Ralstonia*, *Stenotrophomonas*, *Burkholderia* and *Ochrobactrum* have rhizosphere-associated species that can interact with both human and plant hosts. The mechanisms by which these bacteria colonize plants’ rhizosphere and control plants’ pathogens are similar to those responsible for colonization of human cells. Multiple antibiotic resistances are not only common with clinical isolates of these bacterial groups but also with isolates from the rhizosphere. These antibiotic resistances are caused by presence of different antibiotics, high competition and horizontal gene transfer in the rhizosphere. Several PGPR are phylogenetically related to some of the human opportunistic microorganisms and their potential to cause disease can easily be assessed by their ability to survive at 37 °C [167]. The distinctions between pathogens and PGPR can be unveiled via comparative genomics. *Stenotrophomonas maltophilia* and *S. rhizophila* DSM14405T (PGPR) are genomically similar to *S. maltophilia* K279a (a human pathogen) but the former possess genes involved in the breakdown of cell walls of bacteria and plants, Fe sequestration, tolerance to salinity and spermidine synthase production [167]. Irrespective of their origin, both rhizosphere-associated and clinical *Stenotrophomonas* species produce indole-3-acetic acid but environmental species seem to produce more of the phytohormones [168]. Moreover, plant’s innate immune response stimulated by bacterial components like lipopolysaccharide or flagella is similar to the response of human immune system towards pathogens and this systemic acquired resistance was reported by Van Loon and Bakker [169].

Many other N fixing endophytes are also closely related to some human pathogens. In particular, *Klebsiella pneumoniae* Kp342 is an endophytic N-fixer of some agricultural crops and has human pathogenic close relative (strain MGH78578). Strain Kp342 is different from strain MGH78578 since it is able to fix N and lack the genes that encode “global secondary messenger cdi-GMP” known for control of virulent components and biofilm formation. “In total, 4205 proteins (putative orthologues with the average identity of 96%, based on coding sequence prediction) were shared between these two strains, and 1107 proteins were unique to the plant associated Kp342”. Surprisingly, none of the projected coding sequence of Kp342 was similar to that of the already sequenced *Azoarcus* sp. BH72 [99].

In addition, detection of virulence factors in most of these rhizosphere related opportunistic human pathogens is difficult because of the absence of animal models. However, alternative models (often non-mammalian in nature) have been developed for some of these bacteria. For instance, the nematode *Caenorhabditis elegans* and the slime mould fungus *Dictyostelium* discoideum have been used as models to study the pathogenesis of *Pseudomonas*, *Burkholderia Stenotrophomonas* infection [170]. Studies have also shown that similar functions are involved in beneficial associations with plants and virulence in humans. In particular, siderophore uptake is common to plant beneficial bacterial and human pathogens [171]. Dörr et al. [172] suggested that the type IV pili of plant-associated *Azoarcus* sp. BH72 responsible for adhesion on fungal and plant cells have amino acid sequence that is similar to the human pathogens *P. aeruginosa* and *Neisseria gonorrhoeae* indicating that the plant-associated bacterium may be able to cause opportunistic infections in humans.

The category of humans that are susceptible to opportunistic pathogens are the immuno-suppressed patients including AIDS patients, the aged with chronic diseases and those that have been subjected to long-term antibiotic therapy. According to Denton and Kerr [173], long-term antibiotic therapy, prolonged hospitalization, corticosteroid therapy, catheter and admission to intensive care units are some of the risk factors for *Stenotrophomonas* infections. There is even a projection that in future, opportunistic infections will increase because of the increasing numbers of at-risk patients [166]. Therefore, there is an urgent need for management strategies to curb opportunistic pathogens so as to minimize the challenge of environmentally acquired infections.

## 8. Nexus of Rhizobia, Nodule Formation and SYM Pathway

Some members of the Rhizobiales such as *Bradyrhizobium* species and Beta-proteobacteria are capable of forming nodules on the roots of leguminous crops where they transform atmospheric N to ammonia for plant use and gain carbon substrate from the plant in exchange [95,174]. Within the root nodules, the bacteria are confined in specialized cells in the compartments christened symbiosomes and the symbiosome membranes originate from plant cell plasma membrane. The symbiotic interaction between leguminous crops and rhizobia is a good example of prolonged intracellular lifestyle of bacteria within specialized membrane-bound compartments. But the mechanisms by which the symbiotic cells are modified to contain the intracellular bacteria are elusive [175]. Actinobacteria such as species of *Frankia* also form nodules in interaction with plants like Casuarina and Alder. Several bacteria are free living in the environment or may exist as endophytes in plant roots, and similarly have the capacity to fix N [6]. Nodule formation in legumes was first observed approximately 100 million years back [176] long after observing mycorrhizal colonization of plant, indicating that alteration in mycorrhizal pathway gives rise to nodulation. The existence of symbiotic common pathway (SYM pathway) in microbial association with plants raises the question of whether it serves as a route for soil microbiome to gain access to plant root tissues. Oomycetes for instance use this pathway to gain access to plant and cause havoc [177]. Although, mutant strains of rice lacking SYM pathway reveal that certain endophytic microorganisms like *R. leguminosarum* are still able to infect plant roots [103] suggesting that this is not the only pathway available for microorganisms to gain entry into plant tissues. However, plants might be able to detect certain pathogens via the SYM pathway.

## 9. Techniques Use for Investigation of Rhizosphere Microbial Community Structure

Changes in plants’ rhizosphere microbiome caused by the aforementioned rhizosphere modulators can be detected by various techniques. Therefore, researchers can gain insight into the composition and abundance of microorganisms present in the rhizosphere of plants in natural and agricultural ecosystem by using either what is broadly classified as traditional [15] or molecular [4] techniques.

### 9.1. Traditional Techniques

**Figure 2:**
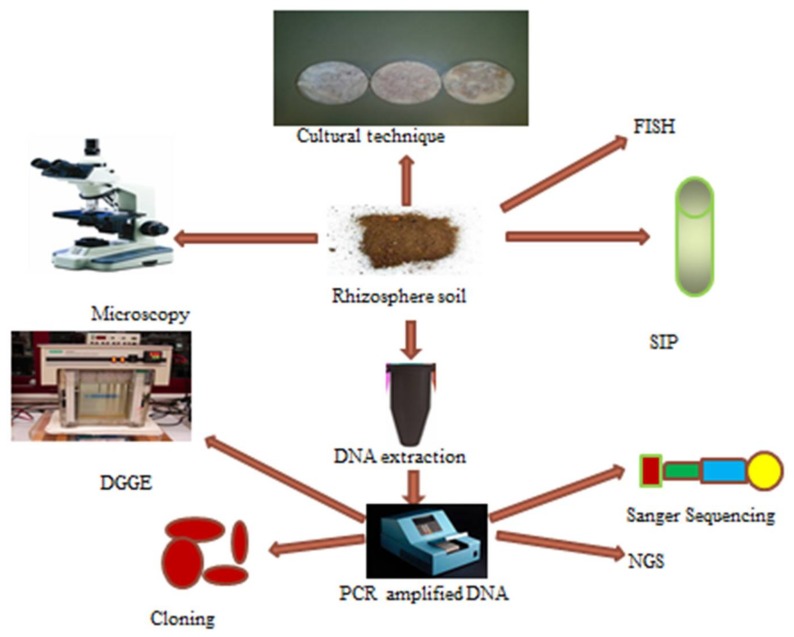
Schematic representation of collective techniques describing culture and un-culture based techniques for the study of rhizosphere microbiomes. DNA-deoxyribonucleic acid, SIP-Stable isotope probing technique, PCR-polymerase chain reaction technique, DGGE-denaturing gradient gel electrophoresis, NGS-next generation sequencing.

Plant microbiome is rich and abundant and they composed of pathogens, endophytes and mutualistic symbionts. In the soil environment, bacterial count could be up to 10^6^–10^7^ cells/cm^2^; this count was obtained by culturing soil samples in nutrient media under different growth conditions (Figure 2).

Plants obtain different nutrients from the soil with the aid of microorganisms in the rhizosphere. These nutrients include majorly N, Fe and P. These elements have the potential to influence plant growth by activating the production of plant growth regulators. Hence, the traditional techniques or culture dependent techniques are routinely used to assay for bacteria responsible for plant growth promotion [178]. These methods involve culturing microorganisms in culture plates or broth to isolate and study PGP traits of PGPR. Some of these PGP traits include siderophores, hydrogen cyanide, phosphate solubilization and exopolysaccharide test. The traditional techniques are also used to isolate and characterize the genetic materials associated with these microorganisms and unfortunately the culture based techniques are not able to capture majority of the unculturable microorganisms in the rhizosphere microbiome [15] as they can only isolate approximately 1% of the entire microbiome in environmental soil samples while the remaining 99% can be studied via molecular techniques or culture independent technologies.

### 9.2. Molecular Techniques

Attempt to profile whole microbiome commenced with the identification and application of 16S RNA gene and the use of PCR for characterization of microorganisms. This had metamorphosed into advanced techniques such as metagenomics use to explore entire microbial community. The drawbacks of these culture independent techniques were recently reviewed [33,179,180] and these technologies involve metagenome sampling, purification, separation, sequencing, analysis of data and interpretations. The sequencing method is rapidly advancing and currently there is NGS or high throughput sequencing (HTS). The HTS techniques comprises of Roche 454 Genome Sequencer, HiSeq 2000, and AB SOLiDTM System [12,16,33,35]. NGS techniques differ from other culture independent techniques like Sanger sequencing in aspects of data analysis and cost. Although NGS has the advantage of making genome sequences handy, data analysis and explanations are currently the challenge associated with the techniques [181]. Moreover, experimental work involving NGS increases the chances of better citation when published. Other molecular techniques involving DNA/RNA stable isotope probing (SIP) and DNA arrays are also used in microbial analysis of environmental samples [33,182]. Indeed, SIP technique (Figure 2) revealed that the root exudates emanating from maize and wheat play a role in shaping microbial community of the soil surrounding the root regions [15]. The microorganisms of the rhizosphere utilizing root exudates were done by analyzing only the denaturing gradient gel electrophoresis (DGGE) profile of ^13^C DNA fixed by plants amended with ^13^CO_2_ soil while microorganisms using organic matter from the soil were evaluated using ^12^C DNA. This investigation demonstrated that some classes of bacteria, e.g., *Sphingobacteriales* and *Myxococcus* could use root exudates emanating from all plants while microorganisms from the order *Sphingomonadales* can utilize carbon substrates of root exudates and soil organic matter.

There is presently transition from the metagenomics approaches to metatranscriptomics techniques. Metatranscriptomics give information about the diversity and functional molecules of the microbial community unlike the metagenomics that only show diversity. It was recently noted that functional diversity of microbiome is probably more dominant in ecological niches than genomic diversity [36,37,183]. Metatranscriptomics methods such as qPCR, help to give information about the functional state of microbiome in the rhizosphere [182]. However, culture independent techniques have a lot of challenges even though the general difficulty of qPCR use for detecting gene expression of microbial community has been surmounted. The challenges entail detecting either ribosomal RNA (rRNA) or messenger RNA (mRNA), accomplishing broader analysis of an environmental RNA pool, designing effective probe and enhancing the performance of sequencing.

Metaproteomics on the other hand, deals with the analysis of protein which involves the extraction of metaproteome from environmental samples and carrying out protein fingerprinting of the extract using mass spectrometry [184,185]. Metatranscriptomics and metaproteomics which are somewhat nascent are confronted with the challenge of sampling and data procurement [184].

Recently, researchers have studied the rhizosphere microbial community of soybean (*G. max* L.) in soil ecosystems in the USA [186]. Yet, much is not known about microbiome associated with the root of most agricultural crops and more research on soil rhizospheric microbiome of crops is therefore needed [59,99,187]. For example, no study has investigated rhizosphere associated microbiota of soybean in field conditions using HTS, even though this is the fourth most produced crops in the globe [188].

Nowadays, virtually all investigations involve molecular analyses which are necessary for detailed characterization of species and investigation of microbial interactions with host plants in the rhizosphere. “Though there are many technical innovations in HTS that lead to insightful and better understanding of the microbiome phylotypes and functions; Dini-Andreote and van Elsas [180] have emphasized its hindrance on testing ecological hypotheses and the current need of a ‘paradigm shift’ from HTS to studies on fundamental questions about yet unexplored plant soil microbiota systems, especially towards phenotypic diversity of rhizospheric microbiome on a spatial and temporal level”.

## 10. Future Direction

“The future trend needs to be in developing genetically modified PGPR over transgenic plants for boosting plant performance, as it is simpler to modify a bacterium than complex higher organisms. Moreover, instead of engineering individual crops, a single, engineered inoculant can be employed for several crops, especially when using a nonspecific genus like *Azospirillum*. PGPR strains development is hampered mainly by the fact that these organisms are sometimes unable to survive harsh environmental conditions, including high concentrations of environmental contaminants, salts, extremities of pH and temperature. Genetic engineering can be used to develop PGPR strains that are effective at low inoculum doses and under a variety of environmental conditions. It is urgent to develop more effective PGPR strains with longer shelf lives to achieve sustainable crop production in dry land production. Recent advances in the fields of microbiology, biotechnology, molecular biology and bioinformatics have opened up the way to identify novel genes involved in drought tolerance. Concepts of micro biotechnology application in agriculture should be employed to isolate indigenous PGPR from the stress affected soils, and screening on the basis of their stress may be useful in rapid selection of efficient strains that could be used as bio-inoculants for crops grown in dry lands [189]”.

## 11. Conclusions

There are myriads of microorganisms including rhizobacteria found in the ecosystem of the rhizosphere and these bacterial interactions with the plants root have been declared beneficial to sustainable agricultural development. This group of bacteria, among other merits can enhance plant development and diminish the occurrence of plant disease. New as well as uncultured microbial candidates in the rhizosphere can better be captured and studied using culture independent techniques which have the potential to analyze broad spectrum of microbial species unlike the culture based techniques. The mechanism by which these microorganisms achieve the mutual benefits on their hosts is not completely comprehended; however, it has been observed that virtually all of their traits enable them to accomplish these benefits. To add to this, rhizospheric microorganisms must be competent in the sense that they should be able to strive within rhizospheric soil that is being influenced by several factors including soil type, plant cultivar and agricultural practices. It is advisable to properly match suitable PGPR with compatible host plant cum environmental condition so as to accomplish better benefits on the plant. This feat will help to reduce the usage of conventional chemical fertilizers as well as pesticides most especially if these microbial inoculants are delivered effectively to the target plant and environment.

## Figures and Tables

**Table 1:** Selected rhizobiome and their contributions towards sustainable agriculture development.

Rhizobacteria Species	Contributions towards Sustainable Agriculture	References
*Azospirillum amazonense*	Enhanced grain yield by increasing dry matter, panicle number and nitrogen content at maturation.	[118]
*Pseudomonas aeruginosa*	Enhanced the remediation capacity of broad bean plants cultivated in soil environment containing oil contaminants. It also helps to control plant pathogens.	[4,119,120]
*Serratia liquefaciens*	Enhanced the remediation capacity of broad bean plants cultivated in soil environment containing oil contaminants.	[2]
*Bradyrhizobium* spp.	Improved nodulation in leguminous plants as well as shoot and root growth. They also enhance plants resistance to drought and production of indole-3-acetic acid	[2,116,117,121]
*Azospirillum* spp.	Enhanced N content in *Vicia sativa*.	[122]
*Rhizobium* spp.	Enhanced significantly the height, pod number and length as well as seed weight of *Vigna mungo* and *Vigna radiate*.	[123]
*Bacillus* spp.	Help plants to develop resistance against pathogens and pest.	[4,120]
*Sinorhizobium meliloti*	Improved biomass diversity in black madic plant that was subjected to copper stress.	[116,124]
*Rhizobium* RL9	Increased lentil plant development, nitrogen content, seed protein content and seed produced under heavy metal stressed environment.	[115]
*Rhizobium* MRPI	Promoted nodule formation, leghaemoglobin concentration, seed protein and seed harvest in pea plant.	[125]

The effects of rhizosphere microorganisms on agronomic crops are highlighted below.
